# Neurodynamic tests for patellofemoral pain syndrome: a pilot study

**DOI:** 10.1186/s12998-019-0242-2

**Published:** 2019-05-08

**Authors:** Kristine Vegstein, Hilde Stendal Robinson, Roar Jensen

**Affiliations:** 1 Manual Therapist/Physiotherapist, Lysaker Manuellterapi, Lysaker Torg 2, Post Box 24, 1324 Lysaker, Norway; 2 Department of Interdisciplinary Health Sciences, Institute of Health and Society, University of Oslo, Postbox 1089, Blindern, 0317 Oslo, Norway; 3 Department of Global Public Health and Primary Care, University of Bergen, Postboks 7804, 5020 Bergen, Norway

**Keywords:** Anterior knee pain, Mechanosensitivity, Femoral nerve test, Femoral slump test, Slump knee bend test, Prone knee bend test

## Abstract

**Background:**

Patellofemoral pain syndrome (PFPS) is a common musculoskeletal disorder. There is little consensus on the etiology, but one explanatory model suggests that PFPS can be caused by referred pain. Neurodynamic tests are used to explore the mechanosensitivity of peripheral nerves, and previous studies have shown a relationship between increased mechanosensitivity and anterior knee pain by using the femoral slump test (FST). Previously the prone knee bend test (PKB) does not appear to have been included. The main purpose of this pilot study was to examine whether there was an identifiable difference in mechanosensitivity between left and right sides that could be identified using both the PKB and FST tests for the femoral nerve in patients with unilateral PFPS.

**Methods:**

This cross-sectional pilot study tested 12 patients with unilateral PFPS for altered mechanosensitivity using both PKB and FST. The pain-free knee was used as a control. The selected test procedures were similar to those clinicians use in everyday practice.

**Results:**

8 and 4 of the 12 patients were found to have increased levels of mechanosensitivity in the PFPS leg using the PKB and FST, respectively. Both tests provoked stronger pain in the leg with PFPS compared with the asymptomatic leg (*p* < 0.05 Wilcoxon Signed Rank Test). The symptoms were more often located in the anterior knee, with structural differentiation by neck flexion appearing to increase the symptoms more when testing the leg with PFPS.

**Conclusions:**

Although the reliability of the tests is unknown and the study sample size was small, the PKB and FST test procedures used in clinical practice appear capable of revealing altered mechanosensitivity in unilateral PFPS patients. The PKB test appears to detect mechanosensitivity in more patients than the FST. We recommend including both tests in future larger blinded controlled studies which should also assess reliability of the tests.

**Trial registration:**

ISRCTN 12473526. Registered 20 May 2015, retrospectively registered.

## Background

Anterior knee pain from patellofemoral pain syndrome (PFPS) is a common musculoskeletal disorder. The main symptom is pain during activity, although there are large variations in the type and level. PFPS tends to affect active young people and can become a long-lasting health issue that prevents patients from living an active life [1]. There is little consensus on the etiology and management [2, 3]. Two models have been presented to explain the cause of PSFS. The first model explains PFPS by biomechanical malalignment, muscle weakness, and overloading of the joint structures and the surrounding patella retinaculum [1, 4–7]. The second model suggests that PFPS can be a result of referred pain from neural structures with nerves from the lumbar plexus causing pain in the anterior part of the knee [8–10].

Several studies have provided new insight into PFPS and support that neurogenic dysfunctions might be involved [3, 11–13]. Elevated levels of local neural markers (substance P and neural growth factor) have been found in the patellar tendon in patients with “jumper’s knee” (a similar condition), indicating the presence of neurogenic inflammation [14]. Dysfunction in the afferent nerves of PFPS patients has been found when using quantitative sensory testing [15]. Immediate pain relief in the knee after manipulation of the back in patients with PSFS has also been reported [16]. These findings can be used to question the pain mechanism in PFPS.

Several clinicians seem to have changed their thinking about PFPS from being a pure “local” mechanical problem to being more the result of sensitization in the nervous system. Due to courses and textbooks on the topic, several therapists have also implemented neurodynamic- tests and treatment for musculoskeletal conditions in both the upper and lower extremities. The purpose of using neurodynamic tests has been to explore the nervous tissue’s ability to tolerate mechanical force and traction. If the test reveals increased mechanosensitivity (i.e., stronger symptoms), it is interpreted as neurogenic dysfunction [9, 10, 17, 18].

Neuorodynamic tests are intended to move neural structures and mechanically apply tension to them. This tension is supposed to arouse a normal positive response that evokes neurogenic symptoms [10]. If the neural structures have altered mechanosensitivity the following abnormal responses have been described: reduced range of movement, stronger pain respons, reproduction of the patients current symptoms when comparing the response in the painful extremity with the asymptomatic extremity [9, 10, 19, 20]. The neurodynamic tests are well known among clinicians but are most often used in patients in which nerve root compressions are suspected. The tests are quick and easy to perform. The prone knee bend test (PKB) and the femoral slump test (FST) (Figs. 2 and 3) are used to identify mid-lumbar nerve root compressions in patients with lower back pain [21, 22]. However, these tests are seldom used to examine patients with anterior knee pain. Nerves from the lumbar-plexus and primarily the femoral nerve innervate the anterior thigh and knee, which means that examination of the femoral nerve could be useful in patients with PFPS. Butler and Shacklock indicate that both tests could be relevant to use for patients with anterior knee pain [9, 10].

Various studies have explored the association between conditions in the lower extremities and dysfunctions of the femoral nerve [20, 23, 24]. Lai et al. explored mechanosensitivity of the femoral nerve, using the FST in patients with anterior knee pain and found the specificity to be above 0.75 [24]. Lin et al. examined neurodynamic responses to the FST in patients with anterior knee pain syndrome. They found increased mechanosensitivity of the femoral nerve in patients with anterior knee pain presenting with a positive FST [20]. Huang et al. found that involving mobilization of the femoral nerve could be beneficial in treatments for PFPS patients with a positive FST [13].

There seems to be a lack of consensus on how to perform and interpret the different neurodynamic tests, as well as how to standardize and grade mechanosensitivity. Furthermore, there are different definitions of a positive test. However, there seems to be agreement that increased mechanosensitivity should be evaluated in each test individually and that the responses should be compared with the contralateral side [10, 17, 24, 25]. Lin et al. compared the neurodynamic responses between subjects with and without anterior knee pain. This study and Lai et al. used testing procedures in laboratory environments that included the use of several straps to maintain the patient’s position. It would be of interest to explore whether easier test procedures similar to what is used in clinical practice could reveal increased mechanosensitivity. To our knowledge, no study on PFPS has used procedures similar to clinical practice and compared the mechanosensitivity in the painful and the asymptomatic leg in patients with unilateral PFPS. Furthermore, we have not been able to find studies that include the PKB test.

The main purpose of this pilot study was to examine whether a side difference in mechanosensitivity could be identified using both the PKB and the FST tests for the femoral nerve in patients with unilateral PFPS and to use the patient’s asymptomatic leg as control. The selected test procedures are similar to what clinicians use in everyday practice. We hypothesized that the responses would be different in the two legs, that there would be a smaller range of motion (ROM) at the onset of the pain/discomfort, stronger pain at the end of the ROM, and that the participants’ anterior knee pain could be reproduced in the PFPS leg. We also hypothesized that there would be a positive structural differentiation in the affected leg, assessed in the FST.

## Methods

The participants in this cross-sectional pilot study were 12 patients with unilateral PFPS who were between 18 and 44 years old. All were recruited from a physiotherapy clinic in primary health care and can be seen as a convenience sample. We identified relevant patients from the clinic’s waiting list, contacted them by telephone, and asked them about participation. An interview and a clinical examination were used to confirm the diagnosis and fulfillment of the inclusion criteria.

The inclusion criteria were: 1) a history of unilateral anterior knee pain located around the patella and lasting for more than 3 months, 2) the ability to participate in normal daily activities, 3) pain at rest, during daily activities (such as knee bends or walking on stairs), or when performing sports or exercise activities, and 4) no symptoms in the other leg, which was used as an asymptomatic control. Patients with known intra-articular disorders, previous surgery or trauma in the legs, or a knee injection during the last 3 months were excluded from participation. During the inclusion process, two patients were excluded: one because of trauma and one with a neurological diagnosis. The Regional Committee for Medical and Health Research Ethics approved the study (2010/819–4).

### Self-reported questionnaire

A pain drawing was used to identify the location of pain, confirm the knee pain, and explore the presence of pain in other areas of the body. Pain intensity over the last 2 weeks was examined by a Visual Analogue Scale (VAS; 0 = no pain, 10 = worst pain). The Norwegian version of the modified Cincinnati Knee Rating System questionnaire (CRS) was used to obtain information about pain and function. The self-administered CRS contains 12 questions about knee pain, swelling, and function, and a sum score is made (0–100; 100 is best). The CRS outcome is evaluated as poor (< 30), fair (30–54), good (55–79), or excellent (> 80). We also calculated two CRS subscores for pain and activity. The maximum in each subscore was 20 points (the best score). The CRS has previously been validated in patients with chronic knee pain [26, 27].

### Functional test

To evaluate the participants’ function, we used a test including both weight bearing and knee bending since these activities commonly aggravate the pain in PFPS patients. Loudon et al. found the step down test to be the most sensitive and reliable test out of five different performance-based tests. Furthermore, they found it to be the only test to discriminate between PFPS patients and controls and reported 100% symmetry between the legs in people with no knee pain [28]*.* Studies have also reported significant lower mean step rates in the affected legs of patients with unilateral pain [3, 28].

**Fig. 1:**
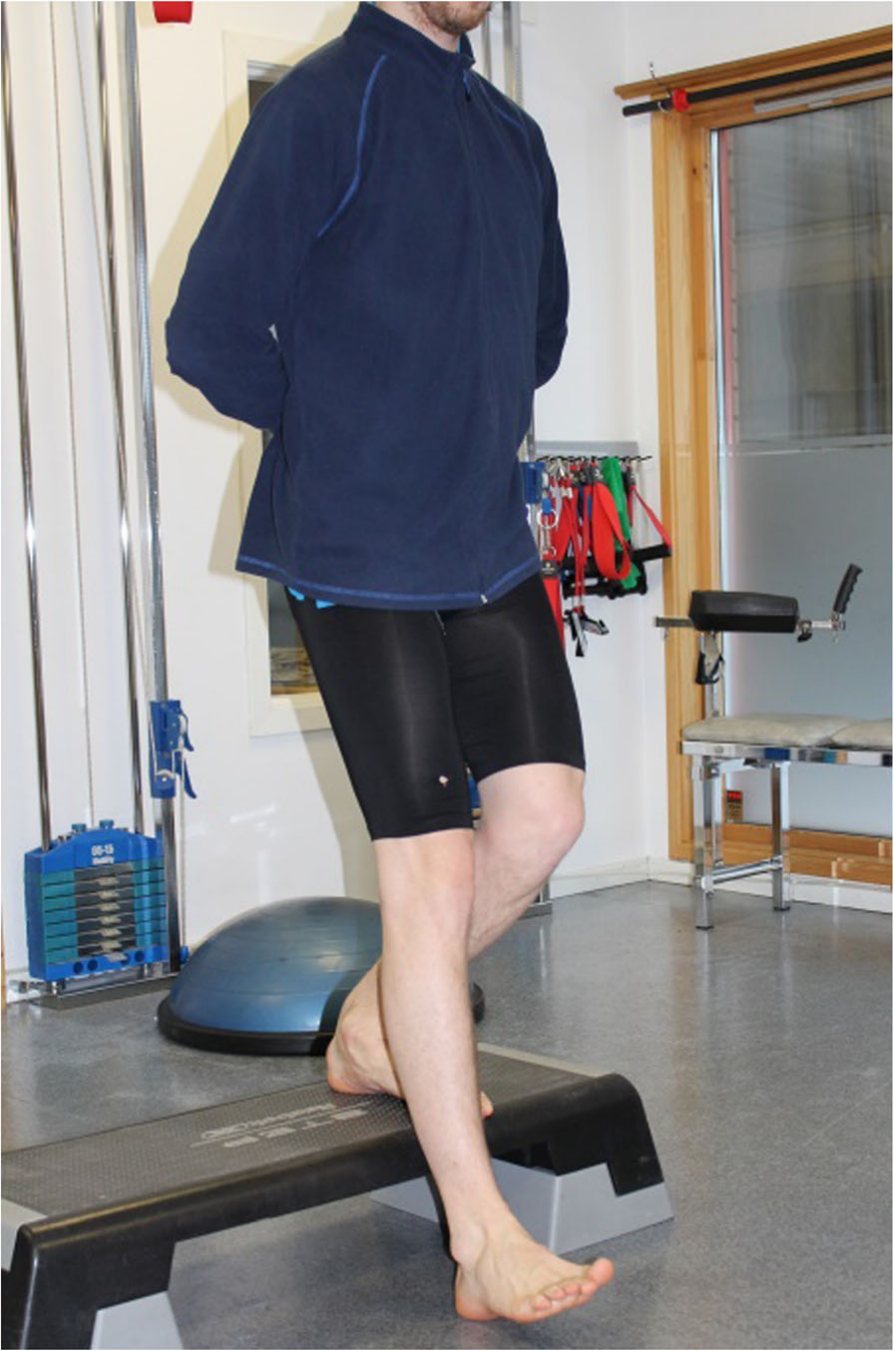
Step down test

We explored whether the test revealed asymmetry between the affected and unaffected leg. The participants performed single leg squats from a 20-cm-high step. They were instructed to go deep enough for the heel on the other leg to touch the floor and then to return to the starting position (Fig. 1). The number of repetitions performed in 30 s was recorded. Both legs were tested, and the difference in number was recorded.

### Neurodynamic tests

**Fig. 2:**
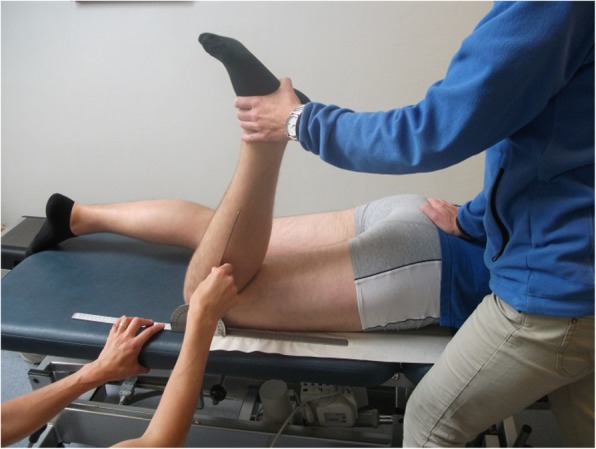
Prone knee bend test

**Fig. 3:**
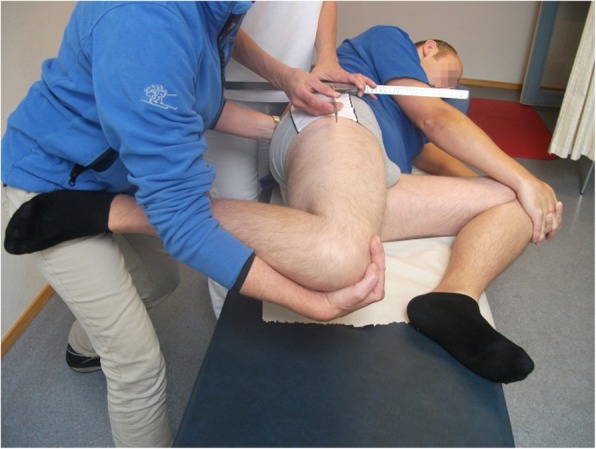
Femoral slump test

We used the PKB and FST to examine the mechanosensitivity of the femoral nerve. The PKB tested the femoral nerve in a prone position (Fig. 2), and the FST tested the femoral nerve in a side-lying position (Fig. 3) [9, 29]. We used test procedures in accordance with the PKB and the slump knee bend test described by Butler [9]. We registered four components with responses: 1) the location of the pain, 2) the ROM (in degrees) at the onset of pain/discomfort, 3) the level of pain (on NPRS) at end of the ROM, and 4) structural differentiation with neck flexion (used during the performance of the FST). A goniometer was used to measure the ROM of the knee and the hip.

To obtain all measurements as positive numbers, we measured the hip-extension ROM in the FST using a different method. We started measuring extension from 90 degrees of hip flexion (i.e., 90 degrees of flexion was defined as the zero point for extension). From this position, hip extension was passively performed until the patient reported pain or discomfort. At this point, we recorded the ROM in degrees. For example, if the patient were able to extend their hip to zero degrees of extension, the measurement would be noted as 90 degrees.

The level of pain was scored with a numeric pain rating scale (NPRS; scores from 0 to 10: no pain to worst pain ever). Each participant was tested twice with a break of 45 min in between. The average of the responses in each leg (ROM and NPRS) in the two examinations was used in the analyses (see Table 1 for a description). The same therapist (therapist 1) examined all participants with the help of one assistant (therapist 2). Therapist 1 had 18 years of clinical practice in musculoskeletal medicine.

**Table 1:** Description of procedures

	Prone knee bend test (PKB)	Femoral slump test (FST)
1	Patient in prone position with hips in neutral position.	Patient in side-lying position. Lower leg held in 90 degrees of hip flexion and stabilized by the patient’s hands. The trunk is in a fully flexed position with the head in a neutral position.
2	Therapist 1 stabilizes the patient’s pelvis with one hand with the other hand placed distally at the tibia. The knee is flexed until the onset of pain/discomfort.	Therapist 1 stands behind the patient at the level of the pelvis. One hand stabilizes the pelvis, and the other supports the knee, which is in 90 degrees of flexion. Patient’s foot is placed on the side of the therapist’s trunk. From a flexed hip position, the therapist then extends the hip until the onset of pain/discomfort.
3	Patient describes the location of the pain/discomfort (knee, thigh, groin, back, other). The response is noted by therapist 2.	Patient describes the location of the pain/discomfort (knee, thigh, groin, back, other). The response is noted by therapist 2.
4	Therapist 2 measures flexion ROM in the knee with a universal goniometer.	Therapist 2 measures extension ROM in the hip with a universal goniometer (starting point/0 degrees of extension is chosen at 90 degrees of hip flexion, and extension is measured from here).
5	Therapist 1 then flexes the knee until the end of the ROM	Therapist 1 then extends the hip until end of the ROM
6	The patient is asked to grade the pain/discomfort on NPRS	The patient is asked to grade the pain/discomfort on NPRS
7		The patient is asked to flex the neck and also to tell if their pain/discomfort is changing. The response is noted.

*PKB* Prone knee bend test

*FST* Femoral slump test

*ROM* Range of motion

*NPRS* Numeric pain rating scale

### Interpretation of responses

#### Pain area

The neurodynamic tests can reproduce pain or discomfort in different bodily areas. If the tests reproduced the patient’s actual pain in the actual area, we considered it as a clinically important response. During the tests, the patients reported pain or discomfort in different areas like the knee, thigh, groin, hip, and back, as well as combinations of pain areas. For the analysis, we categorized the pain locations as symptom areas involving anterior knee pain (scored as 1) and symptom areas not involving the knee (scored as 0).

#### Range of motion (ROM)

The ROM when pain or discomfort was first perceived was measured by a universal goniometer, and the results were compared between the affected and control legs. Various standard errors of measurement have been reported for the universal goniometer (0.52–2.66 degrees in knee flexion and 2.8–3.5 degrees in hip extension). We used a cut-off difference of 3 degrees for the PKB and 4 degrees for the FST [30, 31]. Measurements lower than these values were not interpreted as a difference. Reduced ROM in the affected leg was interpreted as altered mechanosensitivity in the femoral nerve and scored as 1. No difference or increased ROM in the affected leg compared with the control leg was scored 0.

#### Pain level

At the end of the ROM, we compared the pain intensity (NPRS) registered in the affected and the asymptomatic control legs. There is an international consensus that the minimal important change (MIC) on the NPRS in chronic pain patients should be more than 30%. Furthermore, a change of 2 units is defined as “much better,” while a change of 1 unit is “slightly better” [32]. A difference of 1 or more on the NPRS between the two legs was interpreted as a sign of mechanosensitivity. Patients with stronger end-range pain (1 or more) in the affected leg compared to the asymptomatic leg were scored as 1. Less or equal pain in the affected leg compared with the control leg was scored as 0.

#### Structural differentiation

Neck flexion was performed in conjunction with the FST to explore possible changes in pain or discomfort. Structural differentiation where neck flexion increases distal pain or discomfort is generally interpreted as a neurogenic symptom [8–10]. We recorded changes in pain or discomfort caused by neck flexion as either aggravation (scored as 1) or no change (scored as 0).

### Level of increased mechanosensitivity

All components were scored and recorded for both neurodynamic tests, and side-by-side comparison was used to determine whether mechanosensitivity was increased. We decided whether a high level of increased mechanosensitivty was present in the affected leg by using a sum score of all three components for the PKB and all four components for the FST for each patient. Furthermore, we defined a maximum sum score of 3 on the PKB and 4/3 on the FST as a high level of increased mechanosensitivity.

### Statistical analyses

All analyses were performed using SPSS version 18 (IBM Corp., New York, NY). Background data, pain areas, and structural differentiation are presented as frequencies. Pain and function scores are presented with the mean, range, and standard deviations (SD). On the neurodynamic tests, the ROM and pain responses are given as the median and range since the data were skewed. The differences between the affected leg and control legs were tested using the Wilcoxon Signed rank test for continuous variables (NPRS and ROM). The significance level was set as *p* < 0.05.

## Results

There were 12 participants in the study, which comprised 9 women and 3 men with a mean age of 32 (21–42) years. Four participants reported unilateral knee pain only (no pain in other bodily areas), four reported additional pain in the back, and four reported additional pain in other bodily areas (all recorded from the pain drawings). Two of the participants also reported pain in the posterior part of the affected knee. The knee pain was most pronounced during activities such as stair climbing, running, and jumping for all participants, but four participants also reported pain during sitting.

The most predominant characteristics of the quality of the knee pain were “aching” or “sharp.” The duration of the pain varied from 4 months to 5 years. According to the modified CRS, the whole study sample had a fair mean (SD) total score of 52.5 (18). The VAS for the last 14 days and the CRS score showed good correlation (*r* = 0.69, *p* = 0.012). The participants had a lower mean step rate of 19 with the affected leg versus 21 in the asymptomatic leg. Furthermore, 10 of the 12 patients had fewer repetitions with the affected leg compared with the asymptomatic leg. Hence, there was a non-significant difference between the painful knee (PFPS) and asymptomatic knee with a *p*-value of 0.053 (Wilcoxon signed ranked test) (Table 2).

**Table 2:** Description of participants

	Mean (SD)	Range
Pain last 2 weeks (VAS, cm)	3.8 (1.7)	1.0–6.5
Cincinnati knee score total (0–100)	52.5 (18)	24–81
Cincinnati knee score pain (0–20)	8.3 (2.7)	4–12
Cincinnati knee score activity (0–20)	7.8 (5.8)	0–8
Step down test PFPS leg (numbers)	19 (6)	6–28
Step down test control leg (numbers)	21 (6)	7–32

*SD* standard deviation

*VAS* Visual analogue scale

### Results of neurodynamic tests

#### Rom

In the PKB, 9 of the 12 participants had decreased ROM in the affected leg compared to the asymptomatic control leg. The median (range) ROM for the onset of pain or discomfort was 123 (72–133) degrees of knee flexion in the affected leg and 127 (84–140) degrees in the asymptomatic leg. In the FST, only 2 of the 12 participants had decreased ROM in the affected leg compared to the asymptomatic leg. There was no significant difference between groups for the ROM in either test (Tables 3 and 4).

**Table 3:** Prone knee bend test-result

	PKB-ROM			PKB-PAIN LEVEL (NPRS)			PKB-SYMPTOM AREA			TOTAL
ID	PKB -Rom 1	1 = reduced ROM.	PKB-Rom 2	NPRS1	1 = stronger pain	NPRS2	area 1	1 = knee pain	area 2	PKB SUM
1	90	1	112	9	1	7.5	5	1	2	3
2	120.5	1	130	5	1	3.5	5	1	2	3
3	109.5	0	102.5	3	1	2	2	0	7	1
4	133	1	140	1	1	0	1	1	2	3
5	132	0	119	3	0	3	6	1	6	1
6	71.5	1	83.5	3	1	1.5	5	1	2	3
7	115	1	123	3	1	2	6	1	7	3
8	133	1	136	4.5	1	3.5	7	0	7	2
9	125	1	131	3	1	1.5	5	1	2	3
10	130	1	140	2	1	0	5	1	1	3
11	72	1	90	3	1	1	5	1	5	3
12	130	0	130	7	1	5	6	1	5	2
sum		9			11			10		8
median	123 (72–133)		127 (84–140)	3.0*(1.0–9.0)		2.0 (0–7.5)				
*p*-value	*p* = (0.068)			*p* = (0.003)						

ROM-1: range of motion (degrees) when first perceived symptoms in the affected leg

ROM-2: range of motion (degrees) when first perceived symptoms in the asymptomatic leg

1 = reduced ROM: smaller ROM in the affected leg with a difference of 4 degrees or more

NPRS-1: level of pain at end range of motion in the affected leg

NPRS-2: level of pain at end range of motion in the asymptomatic leg

1 = stronger pain: the level of pain in the affected leg was 1 or more on NPRS

Area-1: patients reported pain or discomfort in different areas in the affected leg

Area-2: patients reported pain or discomfort in different areas in the asymptomatic leg

(1 = knee, 2 = thigh, 3 = groin/hip, 4 = back, 5 = knee + 1 area, 6 = knee + 2 areas, 7 = other areas, not knee)

1 = knee pain: reproductions of symptoms involving knee pain in the affected leg (including 1, 5 or 6)

PKB-sum: all patients who scored 3

**Table 4:** Femoral slump test – result

	FST- ROM			FST- PAIN LEVEL			FST - SYMPTOM AREA			STRUCTURAL DIFFERATION POSIIVE		TOTAL
	Rom-1		Rom-2	NPRS-1		NPRS-2	Area-1		Area-2	Leg- 1	Leg −2	FST -SUM
ID		1 = reduced ROM.			1 = stronger pain			1 = knee pain		yes	yes	
1	78	1	90	7	0	7	6	1	5	1	0	3
2	70	1	85	4	1	1	5	1	2	1	0	4
3	96	0	90	5.5	1	3	6	1	6	1	1	3
4	120	0	120	0	0	0	2	0	2	0	0	0
5	92	0	86	4	0	4	2	0	5	1	1	1
6	69	0	65	3.5	0	3.5	5	1	7	1	0	2
7	95	0	95	0	0	0	7	0	7	1	1	1
8	90	0	85	5.5	0	5.5	7	0	7	1	1	1
9	85	0	85	4.5	1	3.5	2	0	2	1	1	2
10	95	0	95	7	0	7	5	1	7	0	0	1
11	85	0	80	4	1	1	5	1	2	1	1	3
12	85	0	85	7	1	6	7	0	7	1	1	2
sum		2			5			6		10	7	4
median	87 (69–120)		86 (65–120)	4.2*(0–7.0)		3.5 (0–7.0)						
*p*-value				(*p* = 0.041)								

ROM-1: range of motion (degrees) when first perceived symptoms in the affected leg

ROM-2: range of motion (degrees) when first perceived symptoms in the asymptomatic leg

1 = reduced ROM: smaller ROM in the affected leg with a difference of 4 degrees or more

NPRS-1: level of pain at end range of motion in the affected leg

NPRS-2: level of pain at end range of motion in the asymptomatic leg

1 = stronger pain: the level of pain in the affected leg was 1 or more on NPRS

Area-1: patients reported pain or discomfort in different areas in the affected leg

Area-2: patients reported pain or discomfort in different areas in the asymptomatic leg

(1 = knee, 2 = thigh, 3 = groin/hip, 4 = back, 5 = knee + 1 area, 6 = knee + 2 areas, 7 = other areas, not knee)

1 = knee pain: reproductions of symptoms involving knee pain in the affected leg (including 1, 5 or 6)

Structural differentiation positive

Yes =1

Leg-1: affected leg

Leg-2: asymptomatic leg

FST-sum: all patients who scored 3 or 4

### Pain area

The PKB provoked anterior knee pain more frequently in the affected leg than the asymptomatic leg. There were 10 participants who reported pain or discomfort involving the anterior knee area when testing the affected leg, whereas 3 reported the involvement of anterior knee pain or discomfort when testing the asymptomatic leg. In the FST, 6 participants reported pain or discomfort involving the anterior knee area when testing the affected leg, whereas 3 reported the involvement of anterior knee pain when testing the asymptomatic leg.

### Pain or discomfort level

Of the 12 patients, 11 reported stronger end-range pain when testing the affected leg compared to the asymptomatic leg in the PKB. The median pain score was 3 (NPRS) in the affected leg and 2 in the unaffected leg. The significant difference was 1 (*p* = 0.003). The difference was also significant in the FST, with a difference in the NPRS score of 0.70 (*p* = 0.041). The median pain score was 4.2 in the affected leg and 3.5 in the unaffected leg.

### Structural differentiation using neck flexion (FST)

When testing the affected leg, 10 of the 12 patients reported an aggravation of the pain during neck flexion, while 2 reported no change. When testing the asymptomatic control leg, 7 participants experienced an aggravation of symptoms with neck flexion, whereas 5 reported no change.

### Sum scores

A high level of increased mechanosensitivity was found in 8 of 12 patients on the PKB test. In these patients all three of the components were positive (provocation of knee pain, reduced ROM, and higher pain score on the affected leg). Two participants had two positive components. A high level of increased mechanosensitivity was found in 4 of 12 patients on the FST test: only one participant had all four components of the FST test positive (provocation of knee pain, reduced ROM, higher pain score, and aggravation of pain when flexing the neck), while three participants had three out of four components positive.

## Discussion

The results revealed somewhat different responses on the two neurodynamic tests when testing the affected and asymptomatic legs. The participants reported stronger pain in the affected leg compared to the asymptomatic leg, particularly when tested with the PKB. Both the PKB and the FST provoked more anterior knee symptoms in the affected leg. Furthermore, all 3 components on the PKB were positive for 8 of the 12 participants (pain area, ROM, pain intensity). Three or four components of the FST were positive for four participants (provocation of knee pain, reduced ROM, higher pain score, and aggravation of pain when flexing the neck). Altogether, these findings can be interpreted as signs of increased mechanosensitivity in the femoral nerve in the leg with PFPS.

To our knowledge, this pilot study is the first to include both the PKB and the FST when examining patients with PFPS, as well as the first to explore these tests in a clinical setting. The PKB is often used in clinical practice, particularly when examining patients with low back pain, but we also consider this test to be relevant for patients with anterior knee pain. In this small sample of patients with unilateral anterior knee pain, the PKB was more often positive in the affected leg and possibly revealed increased mechanosensitivity in more participants than the FST did. This could be interpreted as greater sensitivity in this test, but the result is not clear. Since no study has explored the methodological quality of the tests, our results must be interpreted with caution.

The difference in responses on the two tests could also be due to methodological differences in performing the tests. The FST can be seen as more complex and difficult for the examiner to carry out. The side-lying position makes it more difficult for the examiner to control the spinal flexion and the pelvic stability. According to Shacklock, one common problem is creating sufficient tension in the nervous system through the spinal flexion [10]. This indicates that the PKB can be easier to perform, which might have influenced our results.

It is important to be aware that both tests may induce stress in other tissues like the anterior thigh fascia and muscles and cannot be considered as only testing femoral nerve tension. To exclude the involvement of tissues other than the nerves, the structural differentiation maneuver has been described as essential [9, 10, 19]. Lin et al. considered FST results to be positive when a positive structural differentiation could be found. They also suggested that PFPS patients who presented with positive FST results had altered mechanosensitivity [20]. Since no other study has used both tests, comparisons cannot be made. However, our different findings on the two tests might reveal different aspects of mechanosensitivity, and we recommend implementing both tests when examining PFPS patients.

One major challenge when using neurodynamic tests is that there is no consensus concerning the operational definitions, how to interpret the responses on the different tests, and how to grade the responses. Therefore, we used four different components to define levels of mechanosensitivity (pain area, ROM, pain intensity, and structural differentiation). We compared the affected and asymptomatic legs for each component and interpreted a positive side difference as a sign of altered mechanosensitivity.

The 3 or 4 components are often considered when interpreting the tests in clinical practice but have not been used in studies as far as we know. The components are used mainly in accordance with the description from Butler and Shacklock of how to interpret the neurodynamic tests [9, 10]. Butler and Shacklock also include interpretations of other aspects like the quality of resistance and observation of movement of the pelvis. These components seem to be more difficult to perform, evaluate, and interpret, and we decided not to include them in this pilot study. Since it is not known whether any of the 3 or 4 components are more relevant than the others, we decided to calculate the sum score for each participant. We considered a higher sum as a sign of a higher level of mechanosensitivity. To our knowledge, no other study on neurodynamic testing has used a sum of responses when considering mechanosensitivity.

We found a significant but small mean difference in pain on the NPRS between the legs using the PKB (*p* = 0.003). This difference of 33% exceeds the previously defined MIC, of 30% [33]. The difference was also significant in the FST with difference of the NPRS score of 0.70 (*p* = 0.041), but the MIC did not exceed 30% (17.6%). International consensus literature has suggested that the MIC in NPRS should be 2 [32, 33]. Therefore, the clinical significance of these findings could be questioned.

No statistical difference was found in ROM. However, we did find individual differences between affected and asymptomatic legs in 9 of the 12 patients when using the PKB, and these differences exceeded the standard error of the instrument (the universal goniometer) [30, 31]. The participants reported that both tests reproduced their actual knee symptoms. Reproduction of the patient’s actual symptoms or symptom response in the same region as the patients’ clinical pain has been regarded as relevant [9, 10]. We also found that structural differentiation aggravated the symptoms on the FST in the affected leg more often (in 10 out of 12 patients). However, 7 participants also reported aggravation of the symptoms in the non-affected leg. This finding is also in accordance with results from previous studies [10, 20, 23] and could indicate that the structural differentiation maneuver can aggravate neurogenic symptoms in the asymptomatic (PFPS) limb as well.

The study participants had experienced PFPS for more than 3 months and could be characterized as chronic pain patients [34]. Chronic pain is seen as a complex phenomenon that involves pain mechanisms in the central nervous system, as well as biopsychological factors. Therefore, it is conceivable that central sensitization may have been a contributory factor that might have influenced our results. On the step down test, we observed a lower step down rate (not significant) on the affected leg compared to the control leg in 10 out of 12 patients. This finding contrasts with previous studies reporting significant differences in step down rates between the affected and unaffected legs [3, 28]. This could be due to the low number of participants in the present study or could also indicate participants with better function. However, our study sample reported pain intensity (VAS) and CRS total scores that are in accordance with those of participants in previous studies on PFPS [3, 20, 28].

Our pilot study and previous studies have reported that there might be a neurogenic-dysfunction aspect to PFPS [3, 11–13, 15]. However, there is no gold standard for identifying neurogenic pain or dysfunction, but neurodynamic tests are suggested to be useful for identification [10, 20, 24]. Increased mechanosensitivity in the femoral nerve indicates that PFPS patients could have a neurogenic dysfunction. However, we have not revealed either the location or the cause of the dysfunction. The question remains in regard to whether PFPS is caused by a local peripheral nerve injury (induced by inflammation or entrapments along the femoral nerve) or by inflammation or entrapment of a lumbar nerve root.

Sanchis-Alfonso and Rosello-Sastre found that ischemia in the lateral patellar retinaculum may trigger neural inflammation and could cause anterior knee pain [12, 35]. Furthermore, it is well known that L3 or L4 nerve-root problems can increase mechanosensitivity in the femoral nerve and thus cause anterior-thigh and knee pain [21, 22]. In our study sample, four patients also reported back pain in addition to their primary pain problem (PFPS). Lower back pain is common in the adult population, and this might be a random finding in this sample. On the other hand, the possible relationship between PFPS and lower back pain would be interesting to investigate in futures studies.

### Strength and limitations

The main limitation of the present study is the low number of participants. Hence, the results should be interpreted with caution. However, some of our findings are in accordance with results from previous studies. The use of the unaffected leg as an asymptomatic control could be debatable, but this way of preforming the tests is used in daily clinical practice and should be highly relevant for clinicians. The use of two different tests is a strength, and the different findings might indicate that the tests explore different aspects of mechanosensitivity. Hence, the results indicate that both tests should be used in a larger study.

It has been shown that the act of repeating the tests can influence the results [36]. We tried to reduce this learning effect and the possibility of measurement errors by repeating the tests only twice. We also allowed a break of approximately 45 min between the tests. Moreover, we used the mean responses (ROM and NPRS) on the tests in the two examinations.To our knowledge, no studies have examined the test-retest reliability of the two tests. This can bee seen as a weakness and we recommend that this should be included in future studies. To obtain all measurements as positive numbers, we measured and recorded the hip extension ROM using a different method. Although we found it easier to measure hip extension with this method, it has not previously been used and therfore the lack of reliability can be seen as a weakness. Using one person for all examinations can be seen as a strength with regard to the intratester reliability, but it can also be seen as a weakness since blinding was not possible.

## Conclusion

In this pilot study, including patients with unilateral PFPS, we found differences in responses between the affected and asymptomatic legs using two different neurodynamic tests. The results show that it is possible that these patients had increased mechanosensitivity in the femoral nerve in the affected leg compared with the asymptomatic control leg. Particularly, in the PKB, 8 of the 12 participants had a high level of mechanosensitivity in the affected leg. This finding may indicate that altered mechanosensitivity in the femoral nerve can be part of anterior knee pain. Although the reliability of the tests is unknown and the study sample size was small, the PKB and FST test procedures used in clinical practice appear capable of revealing altered mechanosensitivity in unilateral PFPS patients. Hence, the tests may be worth considering when examining PFPS patients. The neurodynamic tests for femoral pain can easily be included in the standard assessment protocol. However, it is important to measure all components carefully and to interpret the findings with caution. Since this is a pilot study, we recommend that both tests should be included in a study with larger samples and a control group of healthy individuals. Further studies are needed to investigate the neurodynamic tests and the relationship between PFPS and mechanosensitivity.

## Abbreviations

CRS: Cincinnati Knee Rating System questionnaire
HSR: Hilde Stendal Robinson
KV: Kristine Vegstein
MIC: Minimal Important Change
NPRS: Numeric Pain Rating System
PFPS: Patellofemoral pain syndrome
PKB: Prone Knee Bend
QST: Quantitative sensory testing
RJ: Roar Jensen
ROM: Range of Motion
SD: Standard deviations
Test FST: Femoral Slump Test
VAS: Visual Analogue Scale
